# miR‐20a‐5p regulates pulmonary surfactant gene expression in alveolar type II cells

**DOI:** 10.1111/jcmm.14639

**Published:** 2019-09-06

**Authors:** Yongjian Gong, Weidong Xu, Yang Chen, Yun Liu, Yuan Yang, Beibei Wang, Zhitao Lu, Hung‐Chih Lin, Xiaoyu Zhou, Xiaoguang Zhou

**Affiliations:** ^1^ Neonatal Medical Center Children's Hospital of Nanjing Medical University Nanjing China; ^2^ Department of Pediatrics The First People's Hospital of Zhangjiagang City Zhangjiagang City China; ^3^ Department of Pediatrics, Children's Hospital China Medical University Taichung Taiwan; ^4^ School of Chinese Medicine China Medical University Taichung Taiwan

**Keywords:** foetal rat alveolar type II cells, miR‐20a‐5p, phosphatase and tensin homolog, pulmonary surfactant synthesis

## Abstract

MicroRNA (miRNA) critically controls gene expression in many biological processes, including lung growth and pulmonary surfactant biosynthesis. The present study was conducted to investigate whether miR‐20a‐5p had such regulatory functions on alveolar type II (AT‐II) cells. To accomplish this, miR‐20a‐5p–overexpressed and miR‐20a‐5p–inhibited adenoviral vectors were constructed and transfected into cultured AT‐II cells that were isolated from rat foetal lungs of 19 days' gestation. Transfection efficiency was confirmed by observing the fluorescence of green fluorescent protein (GFP) carried by the viral vector, whereas miR‐20a‐5p levels were verified by real‐time PCR. The CCK‐8 assay was used to compare the proliferation ability of AT‐II cells that had over‐ or underexpressed miR‐20a‐5p. The expression of surfactant‐associated proteins (SPs) and phosphatase and tensin homolog (PTEN) was measured by real‐time PCR and Western blotting. In AT‐II cells, transfection resulted in over‐ or under‐regulation of miR‐20a‐5p. While overexpression of miR‐20a‐5p promoted pulmonary surfactant gene expression, its underexpression inhibited it. Consistent with its role in negatively regulating the pulmonary surfactant gene, an opposite pattern was observed for miR‐20a‐5p regulation of PTEN. As a result, when miR‐20a‐5p was rendered overexpressed, PTEN was down‐regulated. By contrast, when miR‐20a‐5p was underexpressed, PTEN was up‐regulated. Neither overexpression nor underexpression of miR‐20a‐5p altered the cell proliferation. miR‐20a‐5p plays no role in proliferation of foetal AT‐II cells but is a critical regulator of surfactant gene expression. The latter appears to be achieved through a regulatory process that implicates expression of PTEN.

## 1 INTRODUCTION

The alveolar type II (AT‐II) epithelial cells at the alveolar horn have highly specialized functions for the synthesis, secretion and reutilization of pulmonary surfactant.^1^ Lack of adequate pulmonary surfactant production could result in the development of respiratory distress syndrome (RDS), one of the major causes of neonatal respiratory failure and death.^2^

MicroRNAs (miRNAs) are small non‐coding RNA molecules found in animal cells that function in RNA silencing and post‐transcriptional regulation of gene expression in many biological processes, such as organism development, cell differentiation and apoptosis, tumorigenesis and inflammatory response.^3^, ^4^ Recent studies have suggested that miRNA molecules might also be involved in the regulation of foetal lung development and maturation, as well as the metabolism of pulmonary surfactant.^5^, ^6^, ^7^, ^8^ For example, Bhaskaran et al^9^ reported that overexpression of miR‐127 significantly decreased terminal bud count, increased terminal and internal bud sizes, and caused unevenness in bud sizes, indicating that miR‐127 may have an important role in foetal lung development. Ventura et al^10^ showed that deletion of miRNA‐17‐92 led to lung hypoplasia. Lu et al^11^ found that the miR‐17‐92 cluster was highly expressed in both the epithelium and the mesenchyme at E11.5 and declined with the lung development. Whether and in what way miRNAs regulate pulmonary surfactant synthesis remains unknown.

Thus, in this present study, we used miRNA microarrays to first detect the differential expression of miRNAs in peripheral blood of premature infants with versus without RDS. We found that miR‐20a‐5p was down‐regulated in RDS premature infants who lacked pulmonary surfactant (PS). The result is consistent with our previous work in rats that showed that the expression of miR‐20a gradually decreased in the late stage of foetal lung development.^12^ Given these results, which were further validated by real‐time qPCR, and in view of the role of PS in RDS, the association between miR‐20a and RDS and the role of miR‐20a in lung development, we further explored the role of miR‐20a‐5p in pulmonary surfactant gene expression.

Bioinformatic analysis has revealed that phosphatase and tensin homolog (PTEN) is one of the target genes for miR‐20a‐5p, which is in keeping with what was reported by Wang et al.^13^, ^14^ PTEN is abundant in lung epithelia where it plays an important role in pulmonary development and pulmonary function.^15^ PTEN inhibition improves wound healing in lung epithelia.^16^, ^17^ In addition, it is well known that PTEN/PI3K/Akt signalling promotes proliferation of lung alveolar progenitor type II cells.^18^

We found that the expression of PTEN increased gradually in the late stage of foetal rat lung development, which was negatively correlated with the expression of miR‐20a‐5p. Furthermore, it was found that miR‐20a‐5p regulated PS and PTEN gene expression in foetal AT‐II cells in a reverse manner. While overexpression of miR‐20a‐5p promoted PS, it inhibited PTEN. By contrast, while underexpression of miR‐20a‐5p inhibited PS, it promoted PTEN. Thus, for the first time we have shown that miR‐20a‐5p plays an important role in regulation of synthesis of pulmonary surfactant. Based upon these findings, we postulate that miR‐20a‐5p might be important in controlling essential developmental and physiological events in the lung, including the synthesis and metabolism of pulmonary surfactant in AT‐II, and may also be involved in the occurrence and development of RDS.

## 2 MATERIALS AND METHODS

### 2.1 Clinical samples

The study was approved by the Ethics Committee of Children's Hospital of Nanjing Medical University, and all the guardians of participants signed an informed consent for participation in this study (approval number: 201701011). Blood samples were obtained from premature infants with RDS <37 weeks' gestational age in 2014. As control, a group of age/sex‐matched premature infants without RDS who had been admitted to the same hospital was also recruited. The diagnosis of RDS was based on the European consensus guidelines updated in 2013.^19^

### 2.2 miRNA array

The miRNA microarray assay was completed by LC Bio in Hangzhou. The main testing steps included sample extraction and quality control, miRNA labelling, miRNA array hybridization, image acquisition and data analysis. The fluorescence intensity of the array was scanned using an GenePix 4000B array scanner (Molecular Devices Co.), and the original image intensity was read using software (Axon). The data were analysed using unsupervised hierarchical clustering (Cluster 3.0) and Tree View analysis (Stanford University, Stanford, CA, USA).

### 2.3 Animals

Sprague Dawley rats were used in this study. These include ten female rats, weight 250‐300 g, and five male rats, weight 300‐400 g in total. All rats were maintained in a specific pathogen‐free animal facility at Animal Center of the Nanjing Medical University.

### 2.4 Isolation, purification and primary culture of foetal AT‐II

Animal use was approved by the Nanjing Medical University Animal Care and Use Committee. Pregnant Sprague Dawley rats were killed with chloral hydrate on day 19 of gestation. Foetal AT‐II cells were isolated using trypsin and collagenase digestions, followed by different centrifugal force and different adherences to remove fibroblasts, as described by Zhu et al^20^ The cells were plated in DMEM with 10% FBS on 24‐well or 6‐well plates and cultured at 37°C and 5% CO2. The nature of the cultures was identified by electron micrograph. Cell viability was >98% as determined by the trypan blue exclusion assay. The purity was >90% as determined by immunohistochemistry with vimentin and fluorescent immunocytochemistry with SP‐C (Figure S1).

### 2.5 Transient transfection

Adenoviral vectors expressing miR‐20a or miR‐20a‐5p inhibitor were used to up‐ or down‐regulate miR‐20a‐5p. The oligonucleotide sequence for miR‐20a was 5′‐CAGCTTCTGTAGCACTAAAGTGCTTATAGTGCAGGTAGTGTGTCGTCATCTACTGCATTACGAGCACTTACAGTACTGCCAGCTG‐3′, whereas the oligonucleotide sequence for miR‐20a‐5p inhibitor was 5′‐CTACCTGCACTATAAGCACTTTA‐3′. The stem‐loop oligonucleotides were synthesized and cloned into an adenovirus‐based vector carrying the green fluorescent protein (GFP) gene (ADV‐U6‐GFP, GenePharma). A universal sequence (ADV1‐NC: 5′‐TTCTCCGAACGTGTCACGT‐3′) was used as a negative control for adenoviral vector.

### 2.6 Proliferation and cell survival assays

Cell viability was assessed using the Cell Counting Kit‐8 assay (Dojindo Laboratories). Cells were seeded in 96‐well plates and transfected with different adenoviruses mentioned above. After 24 hours of incubation, cells were washed and CCK‐8 solution (10 μL) was subsequently added to each well and incubated for 1 hour before the optical density (OD) value at 450‐nm wavelength was recorded. The AT‐II viability at 48 and 72 hours was analysed using the same method, and the experiment was repeated three times.

### 2.7 Reporter assay

The wild‐type pmirGLO‐PTEN‐3′UTR (WT) and the mutant pmirGLO‐PTEN‐3′UTR (MUT) recombinant dual‐luciferase reporter plasmid were designed and synthesized based on the complementary binding sites of miR‐20a‐5p and 3′UTR of PTEN (GenePharma). The 293T cells were divided into four groups, and the two types of recombinant plasmids were cotransfected into 293T cells with miR‐20a‐5p mimic or miR‐20a‐5p negative control, respectively, by Lipofectamine™ 3000 (Invitrogen). After 48 hours, firefly and Renilla luciferase activities were detected by dual‐luciferase reporter assay (Promega Corporation).

### 2.8 RNA extraction and real‐time PCR

The total RNA from AT‐II was extracted with TRIzol (Invitrogen) according to the manufacturer's instructions. The concentration and the purity of the RNA samples were assessed by absorbent density analysis on OD260/OD280.

TaqMan MicroRNA Reverse Transcription Kit (Applied Biosystems) was used to get cDNA sequence of the specific miR‐20a or U6 snRNA. The other cDNA was prepared from 2 μg of total RNA using PrimeScript RT Master Kit (Takara) according to the manufacturer's instructions.

Quantitative real‐time PCR (qRT‐PCR) was performed with SYBR Green PCR Master Mix (Takara), and gene‐specific primers were synthesized according to the published cDNA sequences. PCRs were performed on an ABI 7500 thermal cycler (Applied Biosystems). The primer sequences are shown in Table 1. Relative quantification of gene expression between multiple samples was achieved by normalization against endogenous controls U6 or GAPDH using the 2^−∆∆Ct^ method of quantification.

**Table 1:** Sequences of oligonucleotides used as forward and reverse primers for real‐time RT‐PCR

Gene product	Forward primer	Reverse primer
rno‐miR‐20a‐5p	CGGCGCTAAAGTGCTTATAGTGC	ATCCAGTGCAGGGTCCGAGG
U6	GCTTCGGCAGCACATATACTAAAAT	CGCTTCACGAATTTGCGTGTCAT
SP‐A	AAGGGAGAGCCTGGAGAAAG	GATCCTTGCAAGCTGAGGAC
SP‐B	ATGCACCAGGCCTGCCTTCG	AGCTGGGGCATGTGCCGTTC
SP‐C	GCAGAAACCGCTGCGGGACA	CCCCGAGGCTGTAGGAGACACC
SP‐D	TAACCAACACGTGCACCCTA	CATAGGTCCTGGCAAACCTG
PTEN	TGTCAGGGTGAGCACAAGATA	GCATTTGCAGTATAGAGCGTG

### 2.9 Protein extraction and Western blot analysis

Proteins were extracted from AT‐II cells in RIPA buffer (Beyotime) supplemented with protease inhibitor. The mixed protein from the cell lysates was separated by 10% SDS‐PAGE and transferred electrophoretically to a polyvinylidene fluoride (PVDF) membrane. After soaking with 5% milk in TBS‐T (100 mmol/L Tris‐buffered saline plus 0.1% Tween‐20) for 1 hour at room temperature, the membranes were incubated with the following specific primary antibodies: anti–SP‐A, anti–SP‐B, anti–SP‐C, anti–SP‐D (1:700; Santa Cruz Biotechnology), anti‐PTEN (1:1000; Cell Signaling Technology) and anti–β‐actin (1:1000; CST, USA). After incubating for 12 hours at 4°C, the corresponding goat anti‐rabbit IgG (H + L) secondary antibody (all at a 1:3000; CST, USA) was subsequently applied and immunodetection was achieved by the enhanced chemiluminescence (ECL) system.

### 2.10 Statistical analysis

Data were presented as mean ± SE. from three independent experiments. Statistical analysis was carried out with Student's *t* test (between two means) or one‐way ANOVA test (among more than two means). *P* < .05 was considered as statistically significant difference.

## 3 RESULTS

### 3.1 Differential expression of miR‐20a and PTEN

**Figure 1:**
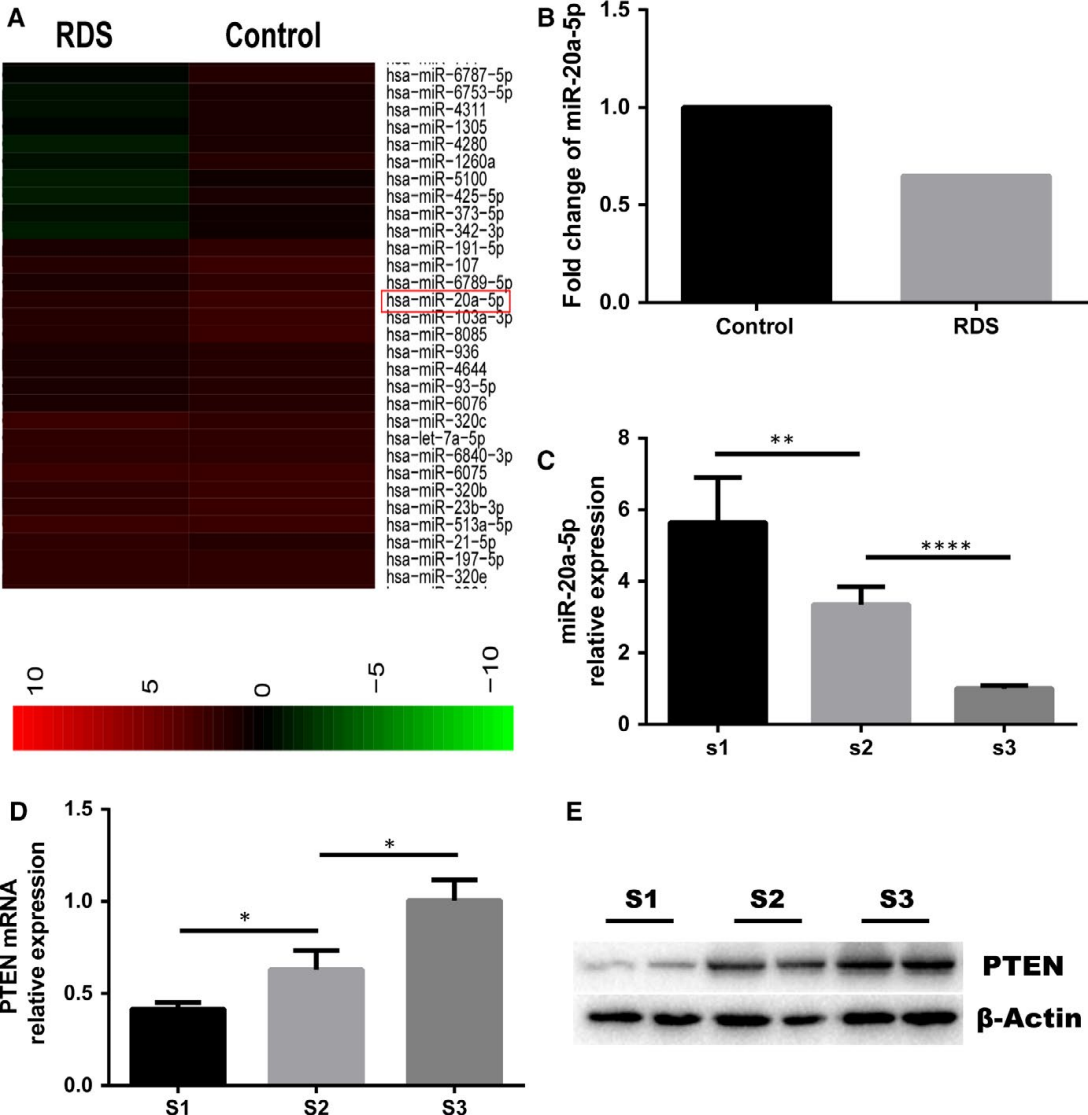
A, Hierarchical clustering of the two groups (RDS vs control). Some distinguishable miRNA expression profiling is observed. Red indicates high relative expression, and green indicates low relative expression. B, The specific fold change of miR‐20a‐5p between the RDS group and control group. C, Expression of miR‐20a‐5p in the foetal lung at three time‐points [S1(E16), S2(E19) and S3(E21)] of rat lung development. D, E, Expression of PTEN gene and protein in the rat foetal lung at three time‐points [S1(E16), S2(E19) and S3(E21)]

We performed miRNA profiling analysis in peripheral blood from premature infants with RDS and without RDS (controls). We observed that miR‐20a was significantly down‐regulated in peripheral blood from infants with RDS relative to infants without RDS. Previously, our group have found that the expression of miR‐20a in rat foetal lungs gradually decreases with lung development.^12^ To validate these results, we measured miR‐20a‐5p expression in the foetal lung at three time‐points of rat lung development [embryonic (E) day 16 (E16), E19, E21] using a conventional real‐time qPCR assay. In keeping with the miRNA array finding, miR‐20a‐5p was down‐regulated during the lung development. In addition, we examined the expression levels of PTEN at these three time‐points in rat lung development and found that its expression gradually increased with lung development. This is in contrast to the expression trend of miR‐20a‐5p (Figure 1).

### 3.2 Up‐regulation/down‐regulation of miR‐20a‐5p in AT‐II cells

**Figure 2:**
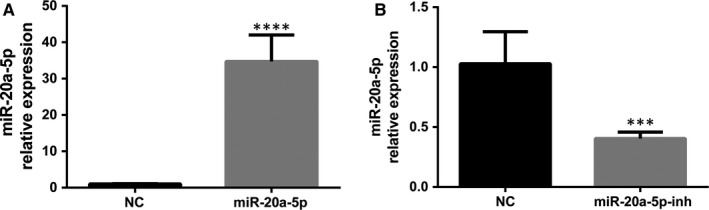
Overexpression and inhibition of miR‐20a‐5p in foetal AT‐II cells. Type II cells were transduced with adenoviruses carrying a universal sequence as negative control (NC), a rat miR‐20a for overexpression, at an MOI of 80, and a rat miR‐20a‐5p inhibitor versus NC at an MOI of 20. At 48 h post‐transduction miR‐20a‐5p levels were measured. A, Overexpression of miR‐20a‐5p (***P < .001). B, Inhibition of miR‐20a‐5p (***P < .001). The miR‐20a‐5p expression levels were normalized to U6 snRNA using equation 2^−∆∆Ct^. Data in the graph are mean ± SE for N = 3

To investigate whether miR‐20a‐5p plays any role in surfactant synthesis, the adenovirus‐expressing miR‐20a was transfected into AT‐II cells that were isolated from rat foetal lungs of 19 days' gestation and cultured. In these experiments, the transfection efficiency was determined by observing the expression of green fluorescent protein (GFP) carried by adenoviral vector. After 48 hours of infection, more than 90% of the cells had positively expressed GFP, showing that the transduction of the adenovirus into AT‐II cells reached over 90% (Figure S2A). To examine whether miR‐20a was overexpressed in AT‐II cells, total RNAs were isolated and mature miR‐20a level was measured by real‐time PCR. As shown in Figure 2A, adenoviral vector for up‐regulation of miR‐20a resulted in a significant higher expression level of miR‐20a compared to negative control. To obtain more evidence that the gene expression of pulmonary surfactant is regulated by miR‐20a‐5p, the adenoviral vector expressing miR‐20a‐5p inhibitor was also used to downgrade miR‐20a‐5p expression. Again, GFP+ cells were more than 90%, and compared with the control group, the expression level of miR‐20a‐5p decreased by 60.6% (Figure 2B; Figure S2B).

### 3.3 miR‐20a has no effect on cell proliferation

**Figure 3:**
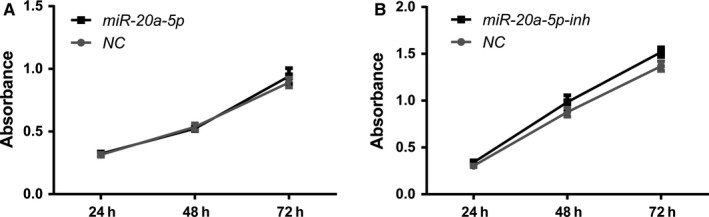
A, Effects of overexpression of miR‐20a‐5p on cell proliferation. B, Effects of inhibition of miR‐20a‐5p on cell proliferation. CCK‐8 result of three groups (Absorbance). Data in the graph are mean ± SE for N = 3. *P* > .05

The CCK‐8 assay was used to detect proliferation ability of AT‐II cells that had overexpressed or underexpressed miR‐20a‐5p. There was no significant difference between the virus control group and the miR‐20a overexpressed or inhibited group in cell proliferation (Figure 3).

### 3.4 Effect of miR‐20a‐5p on the SP gene and protein expression in AT‐II cells

**Figure 4:**
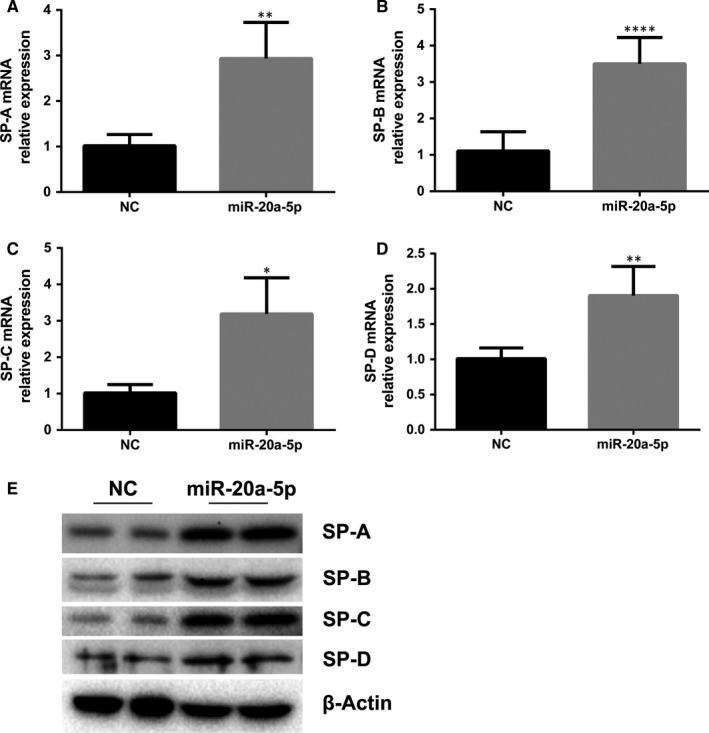
Effects of overexpression of miR‐20a‐5p on surfactant synthesis. AT‐II cells were seeded in plastic dishes and transduced with adenoviruses carrying a universal sequence (NC), a rat miR‐20a (miR‐20a), then cultured for 2 d. Total RNA or protein was extracted. Real‐time PCR was done to determine the mRNA abundance of SP‐A, SP‐B, SP‐C and SP‐D. Western blot was used to detect the abundance of SPs. A, miR‐20a‐5p increased the expression of SP‐A mRNA (**P < .01). B, miR‐20a‐5p increased the expression of SP‐B mRNA (****P < .0001). C, miR‐20a‐5p increased the expression of SP‐C mRNA (*P < .05). D, miR‐20a‐5p increased the expression of SP‐D mRNA (**P < .01). E, Western blots confirm increased SP levels with overexpression of miRNA‐20a‐5p in AT‐II. Left lanes: negative control (NC); right lanes: overexpressed miRNA‐20a‐5p

Having validated the miR‐20a‐5p in AT‐II cells, we next detected the expression of SP‐A, SP‐B, SP‐C and SP‐D genes in these cells, both at RNA and at protein levels, in order to determine whether pulmonary surfactant synthesis in AT‐II cells was affected by miR‐20a‐5p. We assessed RNA expression by real‐time quantitative PCR. SP‐A mRNA, SP‐B mRNA, SP‐C mRNA and SP‐D mRNA levels were found to be significantly increased by 190%, 218%, 215% and 90% in overexpressing miR‐20a AT‐II cells (Figure 4A‐D), in comparison with the virus control group. In addition, we also assessed protein expressions of these SP genes. The proteins were found to be significantly increased as well (Figure 4E).

**Figure 5:**
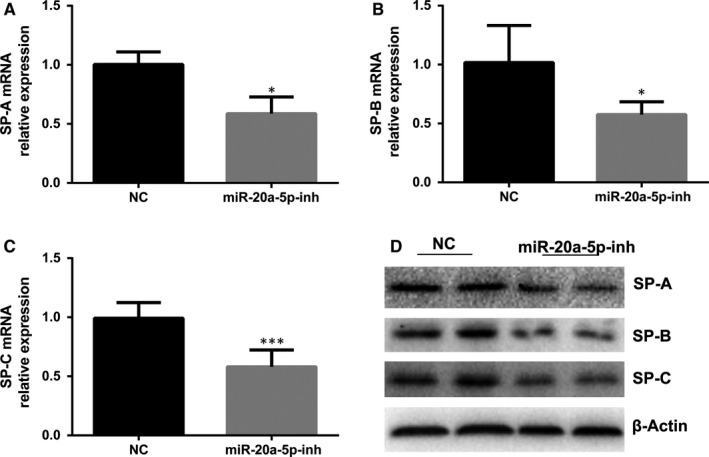
Effects of inhibition of miR‐20a‐5p on surfactant synthesis. A, miR‐20a‐inh decreased the expression of SP‐A mRNA (*P < .05). B, miR‐20a‐inh decreased the expression of SP‐B mRNA (*P < .05). C, miR‐20a‐inh decreased the expression of SP‐C mRNA (***P < .001). D, Western blots confirm decreased SP‐A, SP‐B and SP‐C levels with inhibition of miRNA‐20a‐5p in AT‐II. Left lanes: negative control (NC); right lanes: inhibited miRNA‐20a‐5p

In another set of experiments, we measured SP gene and protein expression after inhibition of miR‐20a‐5p in order to further assess the role that miR‐20a‐5p may play in the regulation of SP. The level of SP‐A, SP‐B and SP‐C, but not SP‐D, decreased when miR‐20a‐5p was inhibited (Figure 5). Thus, it appeared that the effect of miR‐20a silencing was not as obvious as that of overexpression. Alternatively, we failed to detect changes in SP‐D expression after miR‐20a silencing probably because the expression of SP‐D was low.

### 3.5 miR‐20a‐5p targeted expression of PTEN

**Figure 6:**
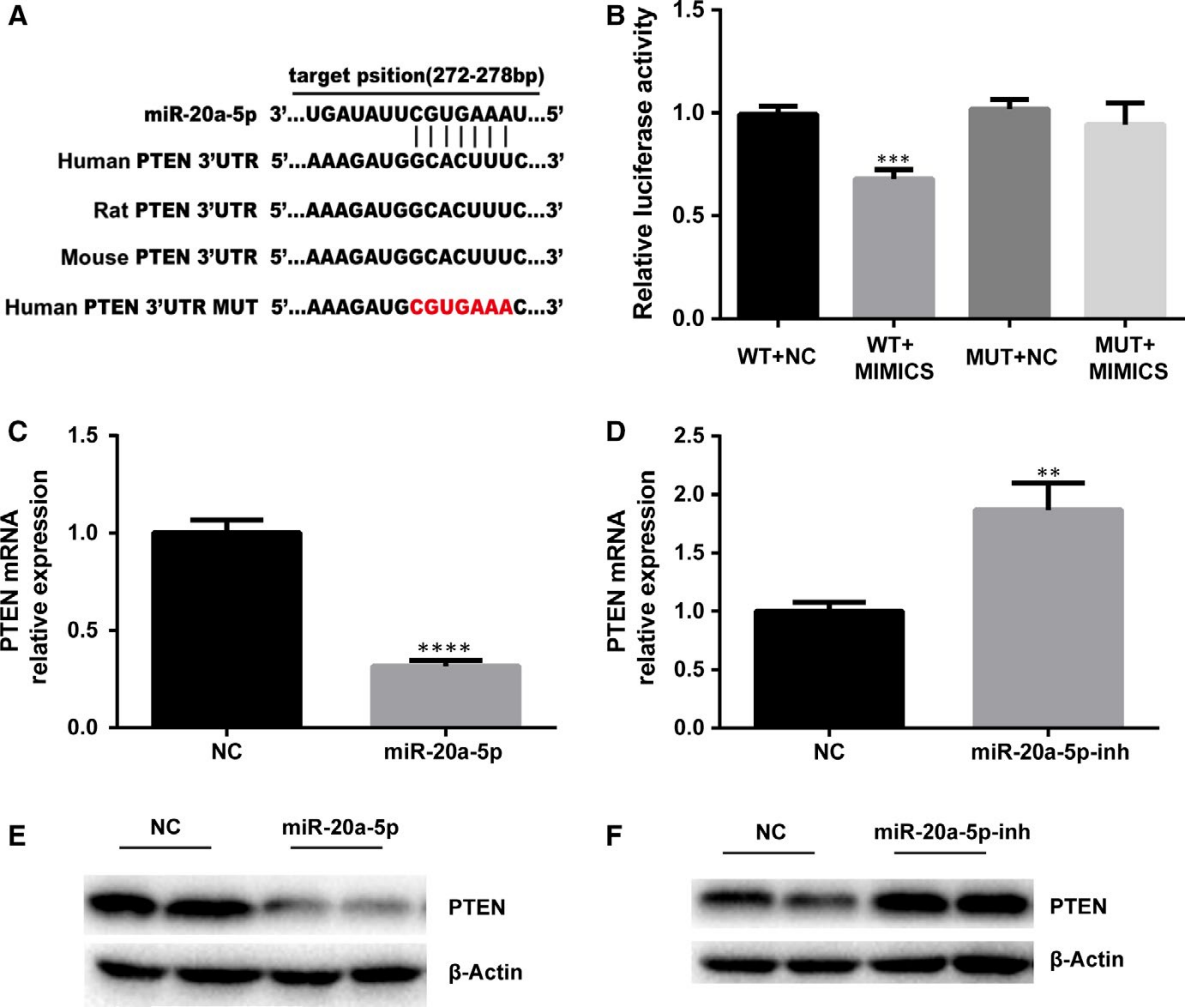
Effects of miR‐20a‐5p on PTEN expression. A, Target position of miR‐20a‐5p at 3′‐UTR of human, rat and mouse PTEN. B, The relative fluorescence activity of PTEN‐WT‐3′UTR and miR‐20a‐5p mimic cotransfected group was decreased significantly comparing with others. mRNA and protein levels were examined using real‐time PCR and Western blot. β‐Actin was used as an internal control. Overexpression of miR‐20a‐5p decreases the PTEN mRNA (C) and protein (E) expression. Inhibition of miR‐20a‐5p increased the PTEN expression (D, F). ****P < .0001, **P < .01

We predicted that miR‐20a targets the PTEN 3′‐UTR. Using TargetScan, we found a perfect base pairing between the seed sequence of miR‐20a and the 3′‐UTR of PTEN mRNA and that these seed sequences were conserved across species, such as humans, rat, and mouse. Dual‐luciferase reporter assay system revealed that the relative luciferase activity of WT‐3′UTR vector and miR‐20a‐5p mimic cotransfected group was decreased by 31.39% comparing with miRNA controls, the differences were statistically significant, while the luciferase activity of MUT‐3′UTR vector was not affected obviously (Figure 6A,B), which is consistent with the results reported in the related literature.^13^, ^14^ Western blot and qRT‐PCR analysis indicated PTEN activity had an inverse correlation with miR‐20a‐5p.

Overexpression of miR‐20a‐5p reduced the expression of PTEN by an average of 68% in comparison with the negative control virus‐transfected cells (Figure 6C). And the level of PTEN increased by 86% following miR‐20a‐5p inhibition in AT‐II cells (Figure 6D). Cells were harvested for Western blot analysis to confirm this effect at the protein level. Densitometric analysis of these protein blots revealed a similar regulatory pattern at the protein level (Figure 6E,F).

## 4 DISCUSSION

Surfactant‐associated proteins (SPs), including SP‐A, SP‐B, SP‐C and SP‐D, account for 10% of PS, but they play a very important role in maintaining the structure, function and metabolism of PS.^21^ Insufficient synthesis of PS by immature foetal lung causes RDS, the main cause of neonatal morbidity and mortality.^2^ In recent years, there has been growing evidence that microRNA is involved in the regulation of foetal lung maturation.^5^, ^6^, ^7^, ^8^ However, currently very little is known about the role of miRNAs in the regulation of pulmonary surfactant synthesis and metabolism.

miRNAs are differentially expressed at different stages of lung development. Our previous work studied miRNA profiling at three different time‐points (E16, E19 and E21) of the developing foetal rat lung and found that miR‐20a was down‐regulated during late lung development.^12^ Similar observation was also made by Lu et al^11^ In this context, we screened the differentially expressed miRNAs in peripheral blood of RDS patients vs non‐RDS controls. We found that miR‐20a expression was indeed significantly down‐regulated in RDS patients (Figure 1A,B). Bioinformatic analysis revealed that the putative targets of miR‐20a‐5p were enriched for several pathways relevant to lung development, including PI3K‐Akt pathway, MAPK pathway and TGF‐b pathways (Figure S3). The large number of predicted targets of miR‐20a‐5p within lung development‐related pathways, coupled with the facts that miR‐20a is highly expressed in the lung and it was down‐regulated in RDS patients, implicates that miR‐20a‐5p could play an important role both in the regulation of the biological process of foetal lung development and in the occurrence and development of RDS.

miR‐20a is a member of the miR‐17~92 cluster, which is the most extensively studied cluster that has an oncogenic function. In addition to its roles in tumorigenesis, miR‐20a has been shown to contribute to hepatic glycogen synthesis by targeting p63,^22^ inhibit TCR‐mediated signalling and cytokine production,^23^ promote the proliferation and migration of human pulmonary artery smooth muscle cells (PASMC) and inhibit their differentiation,^24^ and regulate autophagy in C2C12 cells via targeting ULK1.^25^ Our study shows that in AT‐II cells, miR‐20a‐5p regulates pulmonary surfactant gene expression. We demonstrated that overexpression of miR‐20a‐5p promoted pulmonary surfactant synthesis in AT‐II, as reflected by significantly increased mRNA and protein expression of SP‐A, SP‐B, SP‐C and SP‐D in overexpressing miR‐20a‐5p AT‐II cells. On the contrary, when miR‐20a‐5p was inhibited, down‐regulation of pulmonary surfactant resulted.

As we have known, miRNAs function through targeting their host genes. To further explore the underlying mechanism behind the role of miR‐20a‐5p in regulating pulmonary surfactant synthesis, we searched for the putative target genes of miR‐20a‐5p. Several targets have previously been reported and experimentally validated including CDKN1A, Smad4, p63, EGR2, E2F1, Rab27B, KIF26B and SDC2.^22^, ^26^, ^27^, ^28^, ^29^, ^30^, ^31^ However, none of these targets were found to be associated with the role of miR‐20a‐5p in the regulation of pulmonary surfactant metabolism in AT‐II. Instead, we found that PTEN was a potential target of miR‐20a in AT‐II. This latter is demonstrated by the observations that miR‐20a‐5p levels in foetal lung development were inversely correlated with PTEN levels, that overexpression of miR‐20a‐5p inhibited PTEN expression (Figure 6C,E) and that inhibition of miR‐20a‐5p increased its expression (Figure 6D,F). We also utilized the luciferase reporter gene technique and also concluded that PTEN is the target gene of miR‐20a‐5p. Similar findings were reported by two other research groups, Wang and Zhang.^13^, ^14^

Phosphatase and tensin homolog is relatively abundant in lung epithelia. As a major suppressor of PI3K/Akt signalling pathway and a vital survival pathway in lung parenchymal cells,^32^ PTEN has been implicated in pulmonary development and pulmonary function.^15^, ^16^, ^17^ PI3K/Akt signalling pathway has been widely studied both in vitro and in vivo. The essential biochemical role of PTEN is to remove the D3 phosphate from the inositol ring of phosphoinositides,^33^ so as to inactivate the PI3K signalling axis, thereby preventing the phosphorylation of the downstream intermediate protein kinase, Akt. Recently, more and more studies have demonstrated the role of the PTEN/PI3K/AKT signalling pathway in the lungs. Bao et al^34^ reported that activation of PI3K/Akt axis inhibited Fas‐mediated apoptosis in A549. They found that PTEN suppression significantly reduced the severity of acute lung injury in mice.^15^ Studies by Lai et al and Mihai et al demonstrated that PTEN inhibition facilitated lung epithelial wound repair.^16^, ^17^ Finally, Quan et al^18^ showed that PTEN/PI3K/Akt signalling promoted proliferation of lung alveolar progenitor type II cells. Given these findings, miRNA‐20a‐5p may, at least in part, alter AT‐II biology via inhibiting the PTEN/PI3K/Akt signalling pathways. Consistent with this notion, in the present study we observed that overexpression of miRNA‐20a‐5p in AT‐II improved the synthesis of SP‐A, SP‐B, SP‐C and SP‐D, and concurrently suppressed the expression of PTEN both at mRNA level and at protein level. On the contrary, inhibition of miRNA‐20a‐5p inhibited the expression of SP‐A, SP‐B and SP‐C, and concurrently increased the expression of PTEN. These data support the postulation that miR‐20a‐5p exerts its action via PTEN in AT‐II.

As an oncogene, miR‐20a has been reported to play an important role in cell proliferation, differentiation and apoptosis. Overexpression of miR‐20a promoted gastric cancer cell cycle progression and inhibited cell apoptosis, whereas knockdown of miR‐20a resulted in cell cycle arrest and increased apoptosis.^35^ Studies in mouse suggested that mir‐17‐92 normally promotes the high proliferation and undifferentiated phenotype of lung epithelial progenitor cells.^11^ However, others reported that miR‐17/20a miRNAs function as tumour suppressors by reprogramming tumour cells for NK cell–mediated cytotoxicity.^36^ In this study, we found that the cells with overexpressed or inhibited miR‐20a displayed no significant difference in cell proliferation capacity compared with virus control–treated cells. Our result, coupled with the findings reported by other researchers, suggests that the role of miR‐20a in cell proliferation, differentiation and apoptosis may depend on tissue types to influence protein translation during various cellular processes. It may also depend on their target genes that affect different biological pathways with diverse functions.

Our study was aimed at exploring the regulation of miR‐20a on pulmonary surfactant synthesis in AT‐II cells. There are several shortcomings in this study. First, pulmonary surfactant is composed of phospholipid and pulmonary surfactant protein. As a result of the immaturity of the present technology, we did not detect changes in phospholipid synthesis, but only studied the expression of SP‐A, SP‐B, SP‐C and SP‐D. Second, as a result of the difficulty in extracting primary cells and the limitation of time, we failed to confirm the effects of miR‐20a‐5p in AT‐II cell proliferation by other methods, such as flow cytometry and BrdU incorporation assay. Finally, because of the limitation of time and the availability of technology, we failed to do further experiments such as downstream gene changes in the PTEN/ PI3K/ AKT signalling pathway and functional effect of PTEN on the synthesis of pulmonary surfactant.

In conclusion, our current studies show that in AT‐II cells, overexpressing miR‐20a‐5p promotes pulmonary surfactant synthesis, whereas silencing miR‐20a‐5p inhibits pulmonary surfactant synthesis. We further show that overexpression and underexpression of miR‐20a‐5p cause up‐ and down‐regulation of PTEN. Our findings suggest that miR‐20a is a regulatory molecule capable of modulating the metabolic processes of pulmonary surfactant, and, as such, may have potential role in the treatment of lung‐related diseases such as RDS.

## CONFLICT OF INTEREST

The authors indicate no potential conflicts of interest in this work.

## AUTHOR CONTRIBUTIONS

Author contributions: YJG, WDX and XGZ conceived and designed the experiments; YJG, YC, YL, YY, BBW and ZTL performed the experiments; YJG, WDX and XGZ analysed the data; YJG, WDX and XGZ interpreted the results of experiments; YJG and YC prepared the figures; YJG and WDX drafted the manuscript; HCL, XYZ and XGZ edited and revised the manuscript. All authors read and approved the final manuscript.
