# Supplementary figures and images for: miR‐20a‐5p regulates pulmonary surfactant gene expression in alveolar type II cells
Source: J Cell Mol Med. 2019 Sep 6;23(11):7664–72. doi: 10.1111/jcmm.14639 (PMC6815916; doi:10.1111/jcmm.14639)

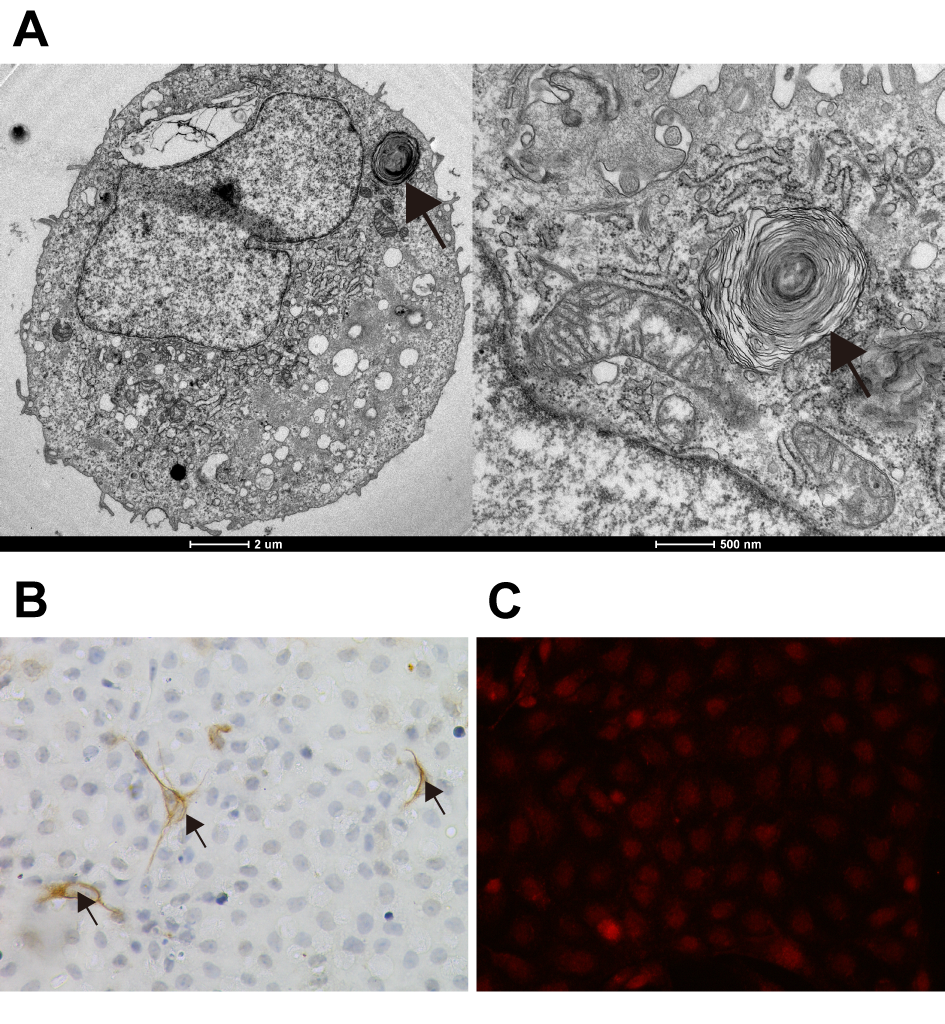

Supplement: Supplementary file 1 [file JCMM-23-7664-s001.tif]

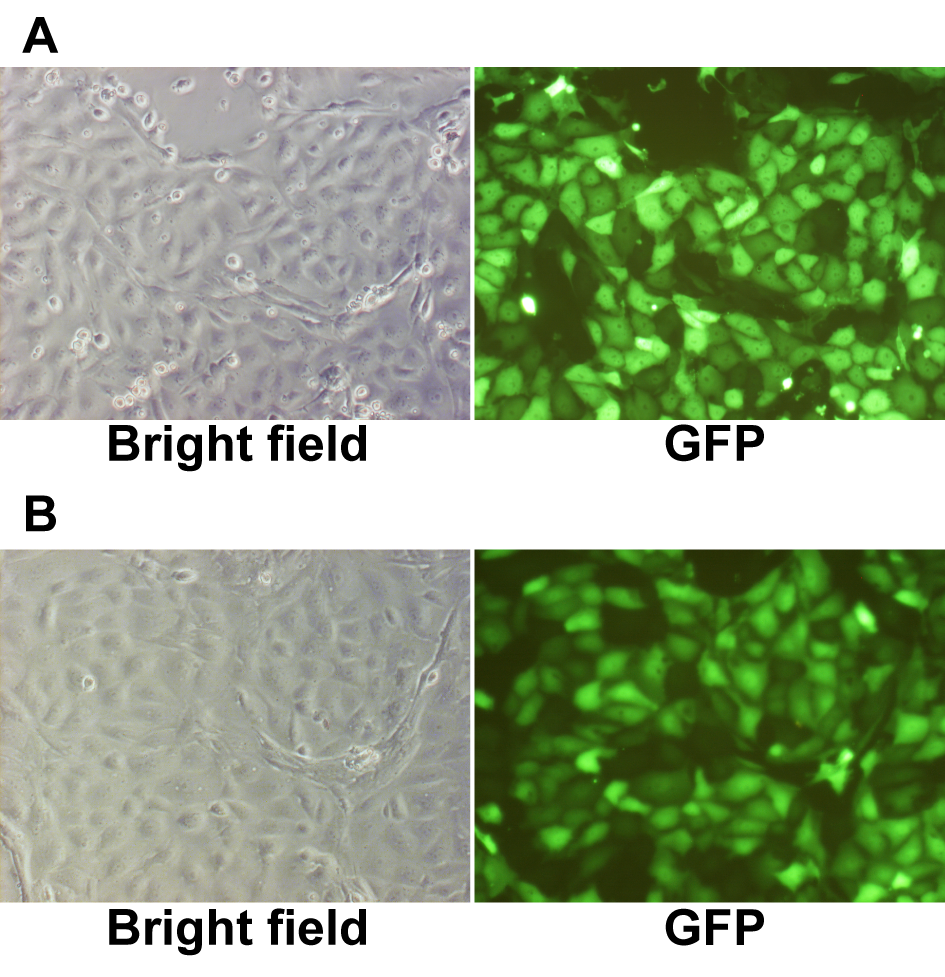

Supplement: Supplementary file 2 [file JCMM-23-7664-s002.tif]

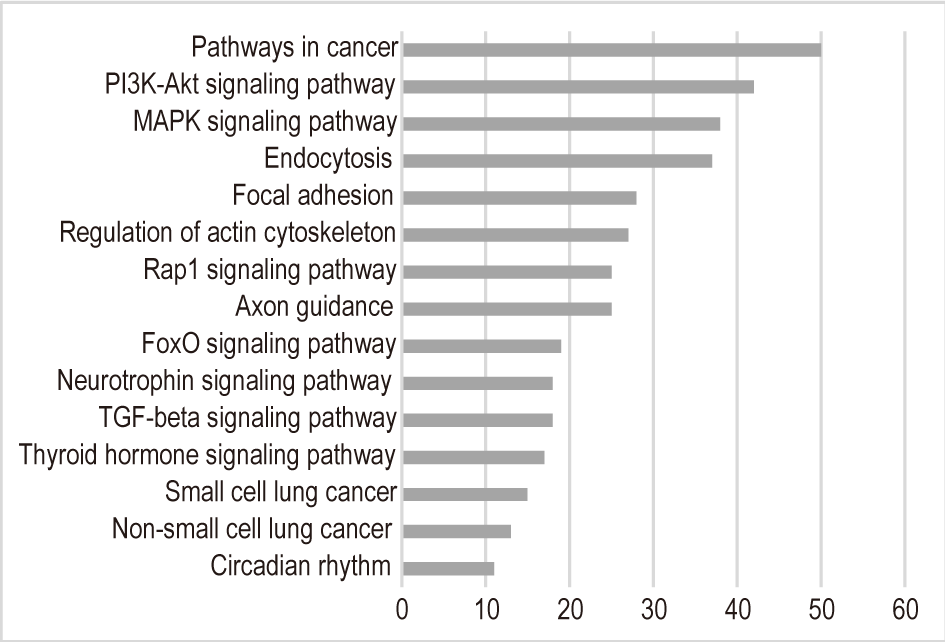

Supplement: Supplementary file 3 [file JCMM-23-7664-s003.tif]
